# Toxicity of orally inhaled drug formulations at the alveolar barrier: parameters for initial biological screening

**DOI:** 10.1080/10717544.2017.1333172

**Published:** 2017-06-02

**Authors:** Eleonore Fröhlich

**Affiliations:** a Center for Medical Research, Medical University of Graz, Graz, Austria;; b Research Center Pharmaceutical Engineering GmbH, Graz, Austria

**Keywords:** Oral inhalation, lung physiology, toxicity, phospholipidosis, alveolar macrophages, dissolution

## Abstract

Oral delivery is the most common mode of systemic drug application. Inhalation is mainly used for local therapy of lung diseases but may also be a promising route for systemic delivery of drugs that have poor oral bioavailability. The thin alveolar barrier enables fast and efficient uptake of many molecules and could deliver small molecules and proteins, which are susceptible to degradation and show poor absorption by oral application. The low rate of biotransformation and proteolytic degradation increases bioavailability of drugs but accumulation of not absorbed material may impair normal lung function. This limitation is more relevant for compounds that should be systematically active because higher doses have to be applied to the lung. The review describes processes that determine absorption of orally inhaled formulations, namely dissolution in the lung lining fluid and uptake and degradation by alveolar epithelial cells and macrophages. Dissolution testing in simulated lung fluid, screening for cytotoxicity and pro-inflammatory action in respiratory cells and study of macrophage morphology, and phagocytosis can help to identify adverse effects of pulmonary formulations.

## 1. Introduction

Oral and enteral routes are the preferred routes for systemic administration of drugs; 38% of all drugs are delivered by sublingual, buccal, oral, and rectal application (Marketsandmarkets, 2017). Pulmonary delivery, by contrast, is used almost exclusively for the treatment of pulmonary diseases. Medication for asthma, chronic obstructive pulmonary disease (COPD), and the combination of both, accounts for 73% of all inhaled medicines (http://www.gilberttechnologies.nl/market-opportunity/). Systemic applications (diabetes, hormone therapy, analgesics, anaphylaxis, influenza, and multiple sclerosis) represent only 18% of the marketed products. Fast absorption by the respiratory epithelium, reduced drug inactivation by first pass metabolization and independence from food uptake make drug delivery by the pulmonary route an attractive option for fast pain relief in migraine, vaccines in pandemic diseases, and emergency applications in labor. Natural peptides are rapidly inactivated by proteases in the gastrointestinal tract but can be applied by the pulmonary route (Patton et al., 2004). An important limitation of pulmonary delivery is the maximal amount of material that can be delivered, and the reduced capacity of the lung to clear not absorbed materials. In local therapy, with the exception of antibiotic treatment of cystic fibrosis with tobramycin (2 × 300 mg (Ramsey et al., 1999)), the required amounts of active pharmaceutical ingredients (APIs) are low. For systemic therapy delivered doses have to be higher in order to achieve effective blood levels. Furthermore, for the treatment of chronic diseases, such as diabetes, APIs have to be applied repeatedly and for longer time periods. This poses the question how formulations are degraded, removed and metabolized at the respiratory epithelium. This is important because accumulation of insoluble material at the respiratory barrier may impair lung function. Respiratory impairment in pulmonary alveolar proteinosis is an extreme example for accumulation of not degraded material at the alveolar barrier (Huaringa & Francis, 2016).

More than 380 medications can induce pulmonary toxicity as side effect and illustrate the particular sensitivity of the lung. The most common manifestation is drug-induced interstitial lung disease (DILD (Schwaiblmair et al., 2012)). The large surface of the lung and the challenge by high levels of oxygen are seen as major contributors to the high vulnerability of lung tissue. Drugs with the highest potential for lung damage are the chemostatic drugs bleomycin, busulfan, and cyclophosphamide, the cardiovascular drugs amiodarone and hydroxymethylglutaryl-CoA reductase inhibitors, the anti-inflammatory drugs aspirin, methotrexate, gold, penicillamine, azathioprine and sulfasalazine, and antimicrobials like nitrofurantoin, amphotericin B, and sulfonamides. Furthermore, biological agents, such as tumor necrosis factor-alpha (TNF-α) blockers, anti-CD20 antibodies, recombinant Interferon-alpha (INF-α), and T-cell antiproliferative agents as well as bromocriptine and cabergoline may cause lung damage. For most of the agents the exact mechanism is not known. The higher accumulation of certain drugs in the lung than in other organs plays a role as well as the lung-specific bioactivation and the reaction to the activation. Due to its high incidence and high mortality of 40–50%, amiodarone-induced lung disease is one of the best-studied diseases. The intracellular accumulation of phospholipids in Μφs and alveolar type II cells is regarded as pathognomonic for DILD. The link of lipid-loaded Μφs to pulmonary toxicity has been confirmed for the antiarrhythmic agent amiodarone, various hydroxymethylglutaryl-CoA reductase inhibitors, and the antidepressant fluoxetine hydrochloride (Dean et al., 1987; Israel-Biet et al., 1987; Lapinsky et al., 1993; Gonzalez-Rothi et al., 1995; Huang et al., 2013). Animal studies suggested the link of the intracellular amiodarone accumulation to decreased pulmonary clearance, while another study reported macrophage activation by the compound (Ferin, 1982; Reasor et al., 1996). In lung toxicity induced by the serotonin reuptake inhibitor venlafaxine also interaction with the drug metabolizing CYP450 system was involved (Ferreira et al., 2014). Furthermore, propellants of pressurized metered dose inhalers and carriers in dry powder inhalers can cause pulmonary irritation (Patil & Sarasija, 2012; Myrdal et al., 2014).

## 2. Defense systems of the lungs

The architecture of the airways presents a barrier for the deposition of inhaled particulates in the respiratory system. Deposition is size-dependent and particles for inhalation are designed for optimal deposition in the respiratory tract, which is maximal (around 40%) for particles of 2–4 μm (Miller, 2000). Once particles get into the lungs they can be removed by acellular systems (mucociliary escalator) and by cellular defense mechanisms (alveolar macrophages, AMs). If compensation systems are exhausted lung overload occurs with morphological lung changes and respiratory dysfunction. It has been speculated that impairment of AMs is the underlying reason (Morrow, 1988). At an amount of 6% phagocytized material decrease in the function of AMs occurs and at 60% cessation of AM function is assumed. The phenomenon of lung overload has been identified in rats and it is still unclear whether it occurs in the same way in humans and how rat effects can be extrapolated to the human situation.

### 2.1 Lung lining fluid

The lung lining fluid represents a protective barrier for the underlying epithelium. In the proximal parts of the lung (large airways), the thickness of the lung lining fluid ranges from 5 to 10 μm and can completely surround inhaled particles (Olsson et al., 2011). The lung lining fluid consists of mucus and particles sticking or being immersed in the mucus are cleared from the lungs by transport via motile elements of bronchial epithelial cells (cilia) to the pharynx. The mechanism is termed mucociliary clearance and most efficient for particles >6 μm (El-Sherbiny et al., 2011). Up to 90% of the inhaled particles are removed within 24 h (Evans & Koo, 2009). The mucus layer measures 2–8 μm in the bronchi and 1.8–3 μm in bronchioles (National Research Council, 1977; Patton & Byron, 2007; Wauthoz & Amighi, 2015). Focal increases of the layer up to 20 times can occur but some small bronchi may completely lack a mucus layer (Hiemstra, 2010). The mucus layer consists of 97% water and 3% solids (mucins, non-mucin proteins, salts, lipids, cellular debris). Mucins are large glycoproteins with charged parts and hydrophobic regions. They form fibers of 3–10 nm in diameter with coverage of typically 20–30 carbohydrates per 100 amino acids. The high content in sialic acid and sulfate creates a strongly negative charge density and renders the polymer rigid by charge repulsion (Lai et al., 2009). Entangled mucins and other mucus constituents with reversible linkage to the polymer, such as lipids and associated proteins (e.g. IgA), primarily form the mucus mesh and also determine the viscosity of mucus. The effective mesh spacing is 30–100 nm (Murty et al., 1984). Hydrophilic APIs are better soluble in mucus than hydrophobic molecules and permeation of the mucus layer is best for uncharged small (<10 nm) APIs (Fröhlich & Roblegg, 2014).

The thickness of the lining fluid in the alveoli (surfactant layer) measures 0.07–0.3 μm and is arranged as a thicker aqueous and a thin lipid layer (National Research Council, 1977; Patton & Byron, 2007; Wauthoz & Amighi, 2015). While respiratory mucus does not show prominent differences to gastrointestinal mucus, the surfactant layer of the alveolus has a unique composition. Surfactant consists of 92% lipids with 41% dipalmitoyl phosphatidylcholine (DPPC) as the main component. Unsaturated phosphatidylcholine contributes with 25%, other lipids with 26% and proteins only with 8% (Parra & Perez-Gil, 2015). Out of these proteins surfactant protein (SP) A has the main contribution with 6%, SP-B and SP-C make up 1% each, and SP-D <0.5% (Green et al., 2010). Analysis of bronchoalveolar lavage fluids showed a mixture of lamellar bodies with different numbers of layers and tubular myelin (Goerke, 1998). Ultrastructural images of the surfactant layer in lung sections indicated various arrangements, partly due to the respiration state in which the lungs were fixed and on the age of the animal. The lining in young animals consists of membranes that show a mesh-like regular structure of up to 200 nm thickness (Walski et al., 2009), while the surfactant layer in senescent rats had an irregular appearance with membranous blebs and absence of a regular myelin-tubular mesh (Tomashefski & Farver, 2008). Modeling data indicate that pure DPPC is interspersed with regions of mixed lipid and protein, which are arranged as bilayers and multilayers (Harishchandra et al., 2010).

**Figure 1.**
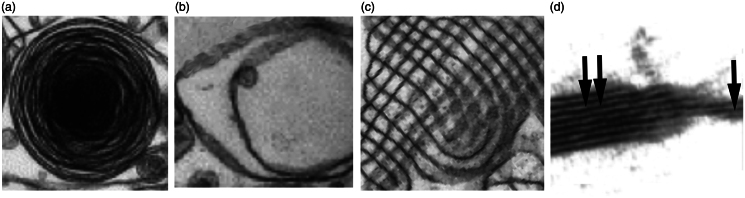
Presentations of surfactant. Alveolar type II cells contain surfactant as intracellular lamellar bodies (a). In the alveolar hypophase lamellar bodies with different numbers of myelin layers (b) and membranes arranged as tubular myelin (c) are seen. On top of the hypophase the surfactant is arranged as multilayer (double arrow) or bilayer (one arrow) (d). Examples were taken from (Schurch et al., 1995; Walski et al., 2009).

The different presentations of surfactant can be described as follows. Surfactant is produced in alveolar type II cells as lamellar inclusion bodies (Figure 1(a)), which are secreted into the alveolar space as lamellar bodies consisting of different numbers of myelin layers (Figure 1(b)) and form a partly crystallized hypophase of tubular myelin (Figure 1(c)). The DPPC-enriched layer on top of the aqueous sub- or hypophase has a double- or multi-layered structure (Figure 1(d)).

### 2.2 Cellular clearance

While particles in the upper parts of the respiratory tract are removed by mucociliary clearance, AMs are responsible for degradation and elimination of API particles that were deposited in the deep lung. AMs are a member of the diverse group of phagocytic cells. Macrophages (Mφs) can polarize into different classes, which are roughly described as inflammatory M1 cells and immune modulatory M2 cells. M2 cells can further polarize into types that play an important role in tumor development and progression (Allavena & Mantovani, 2012). M2a cells induce Th2 response, promote type II inflammation, and help in killing parasites. M2b cells suppress tumor growth, induce Th1 response, and control metastasis. M2c are involved in matrix deposition and tissue remodeling and M2d tumor-associated Mφs promote angiogenesis (Weagel et al., 2015).

Mφs in the lung include AMs and interstitial Mφs (IMs). The latter are located between alveolar epithelium and vascular endothelium and can migrate into the alveolus to become AMs (Boorsma et al., 2013). IMs have a lower capacity for phagocytosis but a higher rate of IL-10 secretion. In addition to M1 and M2 cells, M2-like cells have been identified in the human lung (Satoh et al., 2013). The populations of Mφs undergo specific changes in lung pathologies. M2-like cells decrease, M2 cells increase, and M1 cells first increase and then decrease in asthma. M2-like cells in COPD decrease slightly, while M1 and M2 cells increase. All Mφs in COPD are dysfunctional. In lung fibrosis M2-like, M2, and M1 cells are increased (Boorsma et al., 2013).

In the healthy lung AMs are not activated. They are tethered to alveolar epithelial cells and show slow turnover. Proliferation and survival is regulated by macrophage colony-stimulating factor (M-CSF, CSF-1)/granulocyte macrophage colony-stimulating factor (GM-CSF) (Vlahos & Bozinovski, 2014). These growth factors have slightly different roles by promoting preferentially M1 polarization (GM-CSF) or M2 polarization (M-CSF) (Mia et al., 2014). The attachment to the alveolar epithelium via integrin αvβ6 is important to keep AMs in the quiescent state. Furthermore, tumor growth factor beta (TGF-β), secreted by various cell types in the lung, inhibits AM activation. Stimulation by pathogens induces the switch to the M1 state with secretion of pro-inflammatory cytokines, monocyte recruitment, stimulation of alveolar cells and effector T cells. After removal of the stimulus, reprogramming toward M2 state with abrogation of inflammation, cessation of cell recruitment, apoptosis of inflammatory cells, interaction with regulatory T cells, secretion of lipoxins and resolvins and growth factors for epithelial cell repair, and AT2 to AT1 transition take place (Aggarwal et al., 2014). The secreted proteases activate latent TGF-β and reconstitute the resting state of the AMs (Vlahos & Bozinovski, 2014).

**Figure 2.**
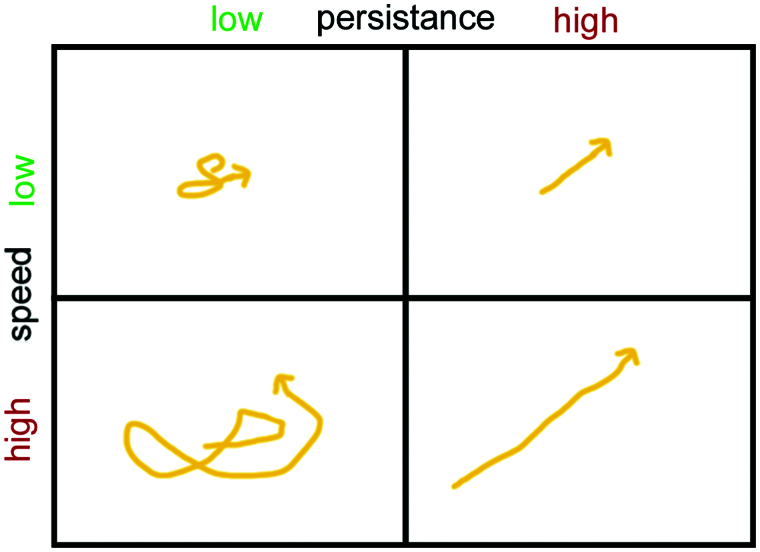
Illustration of cell mobility as result of speed and persistence.

AMs migrate in the alveolar lining layer of around 200 nm thickness (Bastacky et al., 1995). In human lungs obtained from surgery and cadavers 22% of the lung cells were classified as alveolar epithelial cells compared to 3.25% AMs (Crapo et al., 1982). This would correspond to a relative ratio of ∼1:7 but ratios of one AM to forty alveolar epithelial cells in human lungs have also been reported (Crabbe et al., 2011). Fourteen to fifteen AMs have been determined in one human alveole (Geiser, 2010). Although human AMs are more than two times larger than rat and baboons cells, when referred to lung surface, little differences in surface coverage between rodents and humans were detected. On the average, one AM per 18,800 μm^2^ (rodents) or one AM/17,100 μm^2^ (humans) was seen (Miller, 2000; Geiser, 2010). When taking an average size of murine AMs of 121 μm^2^ into account less than one percent of the alveolar surface area is covered by AMs. Based on an alveole surface in mice of 3620 μm^2^ (Knust et al., 2009) and the AM area of 121 μm^2^ this leads to coverage of 3.34% of the alveolar surface (Rodero et al., 2015). This indicates that AMs have to move to ingest and remove deposited particles. Cell mobility is determined by speed (how fast a cell is moving) and persistence (time a cell spends moving in a given direction) (Figure 2 based on (Lauffenburger & Linderman, 1993)).

Persistence time can be influenced for instance by the secretion of chemoattractant. Neutrophilic granulocytes, which possess high speeds of 20 μm/min and low persistence times of 4 min in the normal situation can be guided quickly to inflamed areas by increasing persistence time through secretion of cytokines. In rat AMs speeds of 2 μm/min and persistence times of 30 min have been determined, while human Mφs appear to have lower speeds of 1 μm/min (Bzymek et al., 2016). Studies on murine lung explants determined the velocities of AMs as 2 ± 1.5 μm/min (Rodero et al., 2015).

Calculations by Pollmächer and Figge indicate that a speed of 4 μm/min would be sufficient to find a conidium of *Aspergillus fumigatus* within 6 h before it starts to germ (Pollmacher & Figge, 2015). In the presence of cytokines and with recruitment of additional AMs 2 μm/min would be sufficient. This situation cannot be directly transferred to the situation of inhalation exposure because not one but many particles deposit in one alveole. Other simulations estimate about 5 min as sufficient for AM to patrol across the entire alveolus (Gradon & Podgorski, 1995). High efficacy of AMs for particle removal has been found *in vivo*. Particles of 0.1, 1, and 2 μm size were cleared to 85–90% from the airways (Hofmann & Asgharian, 2002). Non-biodegradable particles of 3–6 μm in diameter were phagocytized by Mφs to more than 80% within 24 h (Geiser, 2002) and biodegradable particles containing growth hormone were removed by Mφs within 24 h to 70% (Patton et al., 1989). In the range of 0.5–4 μm instilled insoluble particles are cleared within 100–200 d from human lungs and lungs of large animal species compared to <50 d in rodent lungs (Kreyling, 1990). The lower extent of clearance suggests that the particle material plays a prominent role in the velocity of the clearance.

### 2.3 Degradation and metabolization

Absorption of substances into the systemic circulation takes place, mainly, at the alveolar epithelium. Alveolar type I (AT1) cells represent only 8.3% of the entire cell population of the lung but cover about 93% of the lung surface (Simon, 1992). They are flat thin cells with long processes and a surface of about 5 μm^2^/cell. Alveolar type II (AT2) cells are about twice as frequent as AT1 cells but the ratio is 1:43 according to surface coverage (Stone et al., 1992). Their main functions include synthesis and secretion of surfactant, xenobiotic metabolism, transepithelial movement of water, and regeneration of AT1 cells (Castranova et al., 1988). Furthermore, AT2 cells are a rich source of antioxidant enzymes; they contain much higher levels of superoxide dismutase, glutathione peroxidase, and glucose-6-phosphate dehydrogenase than AMs and they are more resistant to oxygen exposure than other lung cells, in particular AT1 cells. AT2 cells secrete surfactant lipid and proteins, complement components, prostaglandins, lysozyme, and glutathione.

Both types of alveolar cells can ingest particles up to 200 nm by active mechanisms (endocytosis) (Lankoff et al., 2012). Dissolved material can be absorbed by diffusion and carrier-mediated uptake. Absorption of small hydrophobic molecules is fast and occurs within 1–2 min, for small hydrophilic molecules absorption takes 65 min and the absorption of peptides is still unclear (Liao & Wiedmann, 2003; Patton & Byron, 2007; Mansour et al., 2009).

**Figure 3.**
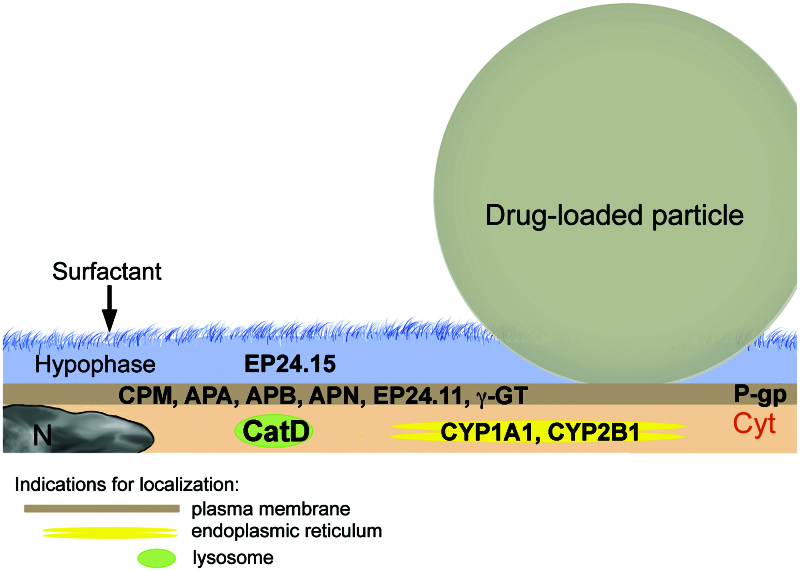
Dissolution and enzymatic degradation of drug-loaded particles by alveolar type I cells (AT1) with indication of the main degrading and metabolizing enzymes. Particle dissolution is slow because particles are only partly immersed in the alveolar lining fluid (hypophase). AT1 cells secrete proteases (EP24.15) in the hypophase. AT1 cells possess various membrane-associated proteases (CPM, APA, APB, APN, EP24.11, and γ-GT), and the lysosomal protease cathepsin D (CatD). The metabolizing enzymes CYP1A1 and CYP2B1 are located in the endoplasmic reticulum. The main transporter MDR-1/P-pg is located at the apical plasma membrane. Abbreviations: CPM: carboxypeptidase M; APA: aminopeptidase A; APB: aminopeptidase B; APN: aminopeptidase N; EP24.11: endopeptidase 24.11; γ-GT: gamma-glutamyltransferase; Cyt: cytoplasm; N: nucleus; P-pg: P-glycoprotein.

APIs are metabolized by the same group of enzymes as in the gastrointestinal tract. The cytochromes P450 (CYPs) constitute the major enzyme family capable of catalyzing the oxidative biotransformation. CYP450 enzymes are so named because they are bound to membranes within a cell (cyto) and contain a colored heme pigment (chrome and P). There are more than 50 CYP450 enzymes, but the CYP1A2, CYP2C9, CYP2C19, CYP2D6, CYP3A4, and CYP3A5 enzymes metabolize 90% of drugs (Lynch & Price, 2007). Gene polymorphism of these enzymes is a major reason for the inter-individual variations in drug levels and has lead to the classification of individuals into poor, intermediate, extensive, and ultrarapid metabolizers. Activities of metabolizing enzymes in the human alveolar epithelium are 1–10% of those in hepatocytes (Somers et al., 2007) and analysis of 10 human lung samples ranked mRNA expression in the following order: CYP1B1, CYP2B6 > CYP2E1 > CYP2C9 > CYP1A1, CYP3A4 and CYP3A5 (Castell et al., 2005). Variations were highest for CYP1A1 followed by CYP2E1 expression, and relatively little variation for the remaining enzymes. Protein expression in human lungs confirmed the gene expression of CYP1A1, CYP1B1, CYP2B6, CYP2E1, and CYP3A5 (Hukkanen et al., 2002). AT1 cells mainly express CYP1A1 and CYP2B1 (McElroy & Kasper, 2004) and AMs contain predominantly CYP3A5 (Hukkanen et al., 2003) (Figure 3). In contrast to rodent lungs, human non-ciliated bronchiolar epithelial cells (Club or Clara cells) possess only low levels of CYP enzymes and do not play a prominent role in pulmonary metabolization of APIs. The ABC (ATP-binding cassette) transporters contribute toward detoxification of xenobiotics by cellular export. Multidrug resistance protein 1/p-glycoprotein (MDR-1/P-gp) is the main exporter for gastrointestinal absorption, biliary, and urinary excretion and regulation of entry into the central nervous system (Vrbanac & Slauter, 2013). MDR-1/P-pg is also highly expressed in the bronchial epithelium and in AMs (van der Deen et al., 2005). AT1 cells express the transporter at the luminal site and in intermediate levels (Campbell et al., 2003), while AT2 cells express MDR-1/P-pg only under oxidative stress and not in the normal condition (Weidauer et al., 2004).

Not only metabolizing enzymes are present at a lower level than in the gastrointestinal tract, also proteolytic activity of the lung is relatively low and partly insufficient to degrade delivered proteins (Okumura et al., 1992). Inhaled superoxide dismutase was found as proteinous aggregates in AMs and in the lung lining fluid (Welty-Wolf et al., 1997). Respiratory epithelial cells contain aminopeptidase N (APN), and dipeptidyl peptidase IV, neutral endopeptidase, and various cathepsins (Cohen et al., 1996; Juillerat-Jeanneret et al., 1997). Aminopeptidases A, B, and N, gamma-glutamyltransferase, endopeptidases 24.11 (enkephalinase), and 24.15 (metalloendopeptidase) are localized at the plasma membrane of AT1 cells (Horalkova et al., 2009). AT2 cells express lysosomal enzymes cathepsin C (dipeptidyl peptidase I), tripeptidyl peptidase I and cathepsin H in their lamellar bodies (Ishii et al., 1991). Out of the lysosomal enzymes, only cathepsin D is expressed by AT1 cells to a greater extent than by AT2 cells (Kasper et al., 1996). The lysosomal enzymes cathepsin B, H, and L are present in both AMs and alveolar epithelial cells (Ishii et al., 1991; Yayoi et al., 2001; Yin et al., 2005). Although Mφs of the lungs contain higher concentrations of proteolytic enzymes than the alveolar cells, they did not play a prominent role in the degradation of insulin. It was reported that degradation of insulin occurred mainly in AT2 cells and only to a low extent in AMs (Finch, 2006). In general, degradation of insulin is lower in the lung than in subcutaneous tissue. Clearance of particles and APIs can further by impaired by external stressors. Smoke, air-borne particulate matter and carbon nanotubes and slowly biodegradable nanoparticles in general decrease AM function, mainly phagocytosis (Fick et al., 1984; Kotani et al., 2000; Renwick et al., 2001; Moss & Wong, 2006; Brown et al., 2007; Hodge et al., 2007; Boyles et al., 2015). The consequence of accumulation of non-biodegradable particles, such as airborne particulate matter, in the deep lung was inflammation leading to fibrous transformation and lung cancer (Bonner, 2007; Winterbottom et al., 2014). Critical evaluation on the pulmonary effects of inhaled human insulin (rDNA origin in Exubera^®^) could not exclude adverse effects of the inhaled insulin in combination with additional stressors of the respiratory system, such as smoking (Seymour, 2006).

In summary, dissolution, degradation, and metabolization of APIs at the alveolar barrier plays an important role for systemic efficacy and pulmonary effects of orally inhaled formulations (Figure 3).

**Figure 4.**
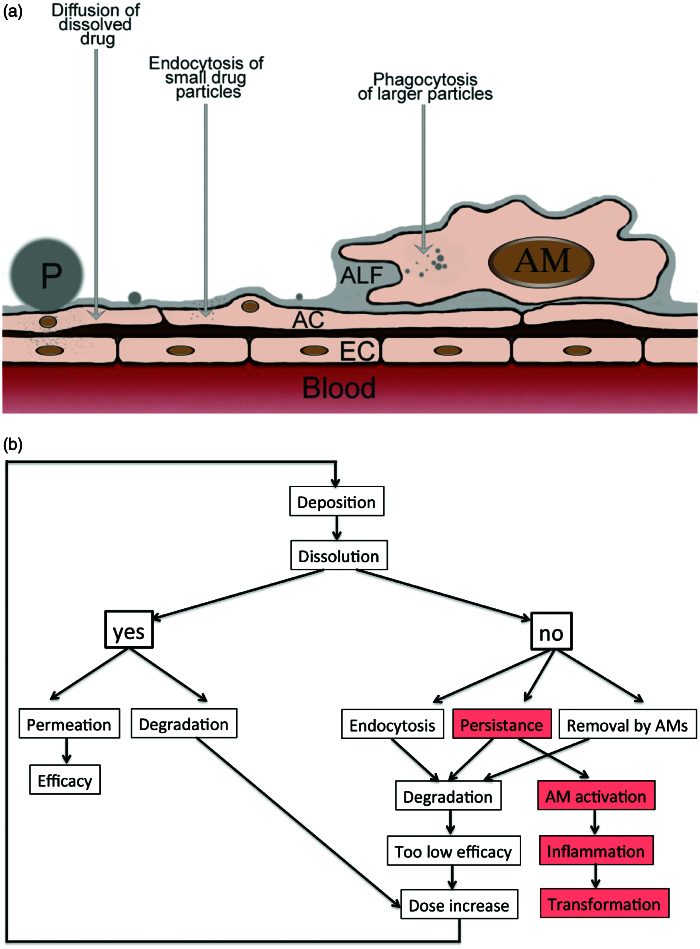
Fate of API formulations in the alveoli as scheme (a) and flow diagram (b). a: Particles can dissolve and diffuse across the alveolar epithelium. Alveolar epithelial cells (ACs) actively ingest small particles while larger particles are phagocytized by AMs. Abbreviations: EC: endothelial cell; P: particle. b: When API particles dissolve fast, either therapeutic levels can be reached in the blood or degradation in alveolar epithelial cells occurs leading to insufficient activity. When dissolution is insufficient small API particles can be taken up by the alveolar epithelial cells and be degraded. AMs can ingest and degrade larger particles that persist at the alveolar barrier. Degradation may result in low systemic drug levels and be counteracted by increase of the applied dose. Persistent particles may also activate AMs and cause inflammation and tissue transformation.

Physiological mechanisms to prevent overload of the lungs with foreign substances and particles comprises the following protective mechanisms. (1) The architecture of the respiratory tract reduces the deposition of particles. (2) Particles deposited on the mucus layer of the larger airways (trachea, bronchi, and bronchioles) are transported to the pharynx to be swallowed. (3) In the smaller parts of the airways (terminal bronchioles, alveoli) non-dissolved API particles are removed by AMs. (4) Particles in the nanosize (<200 nm) can be ingested by alveolar epithelial cells. (5) Degradation and metabolization of APIs occurs at the epithelial membrane, inside epithelial cells and AMs, and by enzymes in the hypophase of the surfactant layer (Figure 4). (6) Dissolved APIs are removed from the respiratory epithelium by permeation (diffusion, carrier-mediated transport, and paracellular transport) across the respiratory epithelium.

## 3*. In vitro* testing

For the preclinical safety studies of formulations for oral inhalation experiments in two species are mandatory and, usually, *in vivo* experiments are started with rodents. By nebulization in the air only few particles will reach the deep lung because rodents are obligatory nose breathers. Application directly to the lung via intratracheal instillation is invasive and bypasses the defense system of the upper respiratory tract. Particles applied by oropharyngeal aspiration or by intratracheal intubation can partly be cleared by mucociliary clearance because solutions are applied at the beginning of the trachea. Since the latter techniques are atraumatic, they are regarded as more physiologic than intratracheal instillation (Fröhlich & Salar-Behzadi, 2014; Ribero et al., 2015). The applied particle dose is influenced by variable loss in the application device. Furthermore, the commonly used volumes of 50–100 μl are much higher than the total amount of epithelial lining fluid in rodents (45–55 μl in rats and 5–15 μl in mice). The application by itself, therefore, may alter the normal lung physiology (Fernandes & Vanbever, 2009).

There are also species differences between rodents and man that have to be taken into account in the interpretation of experimental data from rodents (Table 1). The branching of the airways is dichotomous in humans, while the rat bronchial tree has a monopodial airway branching, which may result in different particle deposition (Hofmann et al., 1989). The lower viscosity of mucus produced by serous glands in rodents compared to the mixed submucosal glands and intraepithelial glands may influence lung physiology. Mucus clearance was reported to be faster in human than in rat lungs (Fernandes & Vanbever, 2009). Further differences regard the cellular composition of the lower respiratory tract and the higher levels of metabolizing enzymes in rat lungs compared to human lungs. Ethoxyresorufin deethylase (EROD, substrate for CYP1A/B) activity, ethoxycoumarin O-deethylase (ECOD, substrate for several CYPs) activity and aryl hydrocarbon hydroxylase (AHH, substrate for CYP1A1) activity per mg of lung tissue were considerably higher in rats than in humans (Carlson, 2008). This can explain the about 10 times higher metabolization of cyclosporine per mg protein in rat compared to human lungs (Vickers et al., 1997). The distribution of specific cell types differs in the way that Club cells are present in the terminal bronchioles of rat lungs but not of human lungs (Harkema, 2000). Serous cells are present in rat lungs, not in human lungs (Hruban, 1984). In order to estimate the availability of APIs in a given formulation at the respiratory barrier *in vitro* tests can be performed in order to identify the most promising formulations for *in vivo* testing and support data from animal experiments. Dissolution testing provides an idea how long non-dissolved particles may remain on the alveolar surface and may be ingested by AMs. A second assessment can identify cellular accumulation and the effect of the API particles on morphology and function of AMs.

**Table 1.** Differences between rodent and human lungs.

Parameter	Rodent	Human
Pleura	Thin with few lymphatic vessels	Thick with many lymphatic vessels
Lung architecture	• Left lung with one lobe • Separation of lobes by little connective tissue • Monopodial airway branching pattern • Smooth muscle fibers do not extent past the bronchiole-alveolar duct junction	• Left lung with two lobes • Separation of lobes by large amount of connective tissue • Dichotomous branching pattern • Smooth muscle fibers extent into the first generation of alveolar ducts
Cell composition	• Serous cells present • Club cells in terminal bronchioles	• No serous cells • No club cells in terminal bronchioles
Mucus clearance	Slower velocity than in humans	Higher velocity than in rats
Metabolization	Higher enzyme content than humans	Lower enzyme content than rats

### 3.1 Dissolution

Due to the low amount of lung lining fluid (∼20 ml) dissolution of APIs in the respiratory tract is slower than in the gastrointestinal tract. It is estimated that the particles used for inhalation (1–3 μm), which are deposited in the alveoles are only partly surrounded by fluid because the height of the alveolar lining fluid has been indicated as 70–300 nm (National Research Council, 1977; Patton & Byron, 2007). The speed of dissolution determines whether particles can be removed by AMs rather than permeate the alveolar barrier. Once the API particles are dissolved log *p* values indicate by which route and how fast absorption across the epithelial layer occurs. Lipophilic drugs with a log *p* > 0 are rapidly absorbed via the transcellular route within ∼1 min. Hydrophilic drugs with a log *p* < 0 are absorbed via the paracellular route with an absorption time of approximately 1 h (Patton et al., 2004). The permeation of poorly soluble inhaled corticoids (fluticasone propionate, mometasone furoate, beclomethasone dipropionate) and antimicrobials (ciprofloxacin betaine, amphotericin B) at high doses is limited by dissolution (Riley et al., 2012). While fluticasone propionate and beclomethasone dipropionate have dissolution times of >5 h, the mean dissolution time of budesonide is 0.1 h suggesting that the former APIs may accumulate at the respiratory barrier. Slow dissolution of drugs might be advantageous for local therapy with anti-inflammatory drugs and with APIs for the treatment of pulmonary infections and pulmonary arterial hypertension. For these indications sustained release is intended and formulations with modified release are being developed (Tiwari et al., 2012; Loira-Pastoriza et al., 2014). For systemic therapy, however, the advantage of persistence at the alveolar epithelium is less clear.

Since no standardized setup and protocols are available for dissolution testing of inhaled formulations, various methods have been published (Table 2).

**Table 2.** Overview on dissolution methods used for orally inhaled formulations.

Particle collection	Dissolution	Reference
Impactor, polycarbonate membrane stainless steel collection base	USP Type II paddle apparatus	Son and McConville (2009); Son et al. (2010)
Powder sealed in membrane	USP Type I basket apparatus	Jaspart et al. (2007)
Impactor, regenerated cellulose membranes	USP Type II paddle apparatus, USP Type IV flow through cell, Franz diffusion cell	May et al. (2012)
Impactor, paper filter	USP Type IV flow-through, Franz diffusion cell, modified Franz cell, beaker method (stirrer)	Wang et al. (2016)
Impactor, glass fiber filter	USP Type IV flow through cell	Davies and Feddah (2003)
Impactor, regenerated cellulose membrane	USP Type IV flow through cell, Franz cell	Jensen et al. (2011)
Impactor, polyvinylidene difluoride membrane	Transwell system	Arora et al. (2010)
Impactor, nitrocellulose membrane	USP Type IV flow through cell, Franz cell	Salama et al. (2008); Salama et al. (2009)

Jaspart et al. described a method where powder was sealed in a filter membrane and immersed within the basket of a Type I dissolution apparatus (Jaspart et al., 2007). This method demonstrated some issues related to the contact area with the powder and appears to be suitable mainly for high-solubility drug products. In most protocols, powders are collected onto polycarbonate membranes or directly onto the stainless steel collection base of the impactors (Son & McConville, 2009; Son et al., 2010). Particle collection on the plates of the impactors may influence the aerodynamic flow profiles of the particles (Riley et al., 2012). In order to avoid this modified cups with a removable impaction insert have been developed. Specified powder cut from each stage is collected on a polycarbonate membrane and covered with a membrane of the same material (Son et al., 2010). The drug contained within the two membranes is clamped into the holder and immersed into the dissolution vessel, for instance a standard USP Method 2 apparatus containing 300 ml of dissolution fluid. Smaller particles on the impactor stage with a cutoff diameter of 0.94 μm have a large surface area to volume ratio and are expected to exhibit a faster dissolution rate in accordance with the standard Noyes–Whitney model (Son et al., 2010). Available studies used filter pore sizes of 0.45 μm and it has been reported that orientation of the filters had an impact on the dissolution rate (Jensen et al., 2011). Furthermore, the dissolution rate was dependent on drug loading (Son & McConville, 2009; Arora et al., 2010). The role of the membrane material has been studied systematically in Franz cells using ibuprofen (MW 206.4, log *p* 3.5, pKa 4.5) as the model drug. Membrane materials included regenerated cellulose, cellulose esters, cellulose nitrate, polyacrylonitrile, polyamide (nylon), polyethersulfone, polysulfone, polycarbonate, polypropylene, and polydimethylsiloxane. The membranes differed also in other parameters, thickness (10 μm 400 μm), pore size (0.05–0.45), porosity (8–84), and tortuosity (1–1.5). The authors concluded that the ideal high flux membrane for formulation analysis should have high porosity (> 60%), tortuosity of 1, and be relatively thin (∼10 μm) (Ng et al., 2010). A systematic study of various dissolution setup systems (paddle apparatus with membrane holder, flow through cell or Franz diffusion cell) identified the paddle apparatus as most appropriate to discriminate between good and poorly soluble substances (May et al., 2014). The main concern for the prediction of *in vivo* dissolution is the fact that the dissolution volume in the paddle apparatus (300 ml) is much higher than the amount of lung lining fluid, which has been indicated in most publications as 12–26 ml (Fröhlich et al., 2016). Another approach to determine dissolution is the combined chemical and microscopical evaluation of particle dissolution commercialized as DissolvIT^®^. The dissolution cell is positioned on top on an inverted microscope and perfused in flow-past configuration. The cell consists of an injection-molded polycarbonate cell with a porous polycarbonate membrane. There are currently too few data obtained with this system to conclude whether this system has advantages over the other setups (Börjel et al., 2014).

Several recipes for simulated lung fluid (SLF) are available in the literature (Table 3).

**Table 3.** Composition of simulated lung fluids used for dissolution studies.

Composition	Gamble solution 1	Gamble solution 2	Gamble solution 3	Gamble solution 4	Gamble solution 5	Modified Gamble solution	Pseudo alveolar fluid	Simulated lung fluid	Artificial interstitial fluid	Synthetic serum	SELF	SLF (mM)
MgCl_2_⋅6H_2_O (mg L^−1^)	95	203	203				212	212	203		200	
NH_4_Cl (mg L^−1^)				535	118	5300				535		10
NaCl (mg L^−1^)	6019	6019	6019	6786	6400	6800	6415	6400	6193	6786	6020	11,6
KCl (mg L^−1^)	298											
CaCl_2_ (mg L^−1^)				22						22		
CaCl_2_⋅2H_2_O (mg L^−1^)	368	368	368		225	290	255	255	368		256	0,2
Na_2_SO_4_ (mg L^−1^)	63	71	71				79		71		72	
H_2_SO_4_ (mg L^−1^)				45		510				45		0,5
Na_2_SO_4_⋅10H_2_O (mg L^−1^)								179				
Na_2_HPO_4_ (mg L^−1^)	126				150		148	148	142		150	
NaH_2_PO_4_ (mg L^−1^)		142	142	144						144		
NaH_2_PO_4_⋅H_2_O (mg L^−1^)						1700						1,2
H_3_PO_4_ (mg L^−1^)						1200						
NaHCO_3_ (mg L^−1^)	2604	2604	2604	2268	2700	2300	2703	2700	2604	2268	2700	27
Na_2_CO_3_ (mg L^−1^)						630						
NaHC_4_H_4_O_6_⋅2H_2_O (sodium hydrogen tartrate dihydrate) (mg L^−1^)							180	180				
H_2_C_6_H_5_O_7_Na⋅2H_2_O (sodium dihydrogen citrate dihydrate) (mg L^−1^)	97	97	97				153	153				0,2
CH_3_CHOHCOONa (sodium citrate) (mg L^−1^)	574			52	160		175			52		
Citric acid⋅H_2_O (mg L ^1^)						420						
NaOCOCOCH_3_ (sodium pyruvate) (mg L^−1^)							0,72	172				
NH_2_CH_2_COOH (glycine) (Gly) (mg L^− 1^)				375	190	450	118	118		450	376	5
L-Cysteine (C_3_H_7_NO_2_S) (mg L^−1^)				121							122	1
DPPC (dipalmitoyl phosphatidyl choline) (C_40_H_80_NO_8_P) (mg L^−1^)			200		200				200		100	
CH_3_COONa⋅3H_2_O (sodium acetate trihydrate) (mg L^−1^)		953	953						952			
Sodium acetate (CH_3_COONa) (mg L^−1^)						580						
HOC (COONa) (CH_2_COONa)_2_⋅2H_2_O (sodium citrate dihydrate) (mg L^−1^)						590			97			
C_3_H_5_NaO_3_ (sodium lactate) (mg L^−1^)								290				
KCl (mg L^−1^)		298	298						298		298	
Potassium hydrogen phthalate (C_8_H_5_KO_4_) (mg L^−1^)						200						
C_14_H_23_N_3_O_10_ (DTPA) (pentetic acid) (mg L^−1^)				79								0,2
C_21_H_38_NCl (ABDAC) (mg L^−1^) (benzalkonium chloride)				50								50
Ascorbic acid (mg L^−1^)											18	
Uric acid (mg L^−1^)											16	
Glutathione (mg L^−1^)											30	
Albumin (mg L^−1^)											260	
Mucin (mg L^−1^)											500	
pH (adjustment with HCl)	7.4			7.3		7.4	7.6		7.4	7.3	7.4	
Reference	Colombo et al. (2008)	Moss (1979); Ungaro et al. (2009)	Yang et al. (2008)	Wragg and Klinck (2007)	Julien et al. (2011)	Gray et al. (2010)	Takaya et al. (2006)	Taunton et al. (2010)	Stopford et al. (2003)	Kanapilly et al. (1973)	Boisa et al. (2014)	Cheng et al. (1997)

Gamble’s solution, at acidic and neutral pH, has been developed for the testing of environmental particles (for instance: (Wragg & Klinck, 2007; Colombo et al., 2008; Sdraulig et al., 2008; Gray et al., 2010; Julien et al., 2011)). The acidic pH should mimic the situation in lysosomes of AMs and IMs, while the neutral pH should represent the interstitial fluid. Similar recipes were later employed for the testing of pulmonary drug delivery systems (examples: (Taylor et al., 2006; Yang et al., 2008; Ungaro et al., 2009)). Since alveolar lining fluid *in vivo* contains a high amount of DPPC (see section 2.1), the use of buffer + DPPC has been tested. Addition of 0.02% DPPC increased the dissolution of inhaled corticosteroids (Davies & Feddah, 2003). The positive value of adding DPPC to the dissolution solution is not unanimously accepted. It has been postulated that the formation of liposome aggregates may hinder the passage of drug through the membranes. To produce DPPC-liposomes for pulmonary delivery, Cook et al. used hydration of dry films produced by chloroform:methanol evaporation and sequential extrusion through 1, 0.4, and 0.2 μm membranes. The resultant liposomes had sizes of 168 ± 4.2 nm (Cook et al., 2005). In another protocol DPPC liposomes in SLF were prepared by sonication instead of extrusion without indication of size distribution (Son et al., 2010). The influence of the preparation method on the observed dissolution profiles is unknown. In addition to salts, SLF in some studies contained antioxidants (Wragg & Klinck, 2007) or DPPC (Stopford et al., 2003; Yang et al., 2008; Julien et al., 2011) and mucin plus albumin (Boisa et al., 2014). Llinas et al. screened dissolution of APIs with different logP, pKa, and intrinsic solubility in buffers containing either 0.5% sodium dodecyl sulfate (SDS), 0.025% DPPC or 0.003% Curosurf^®^ (porcine lung surfactant extract). There was no SLF composition that provided optimal dissolution for all APIs. The main conclusions were that no differences in the dissolution between the SLFs was seen for hydrophilic APIs, that hydrophobic APIs profited from addition of SDS, that the effect of DPPC was similar to the natural surfactant Curosurf^®^, and that high ionic strength of the SLF decreased API solubility (Llinas et al., 2014). The evaluation of the best SLF should also take compatibility with cells into account. Determination of permeability as apparent permeability (P_app_) value is a common parameter in formulation development. To create more physiologically relevant conditions, the routine protocol can be modified in the way that compounds are applied in physiological solutions, such as fasted simulated intestinal fluid (FaSSIF) for oral compounds (Mercuri et al., 2016). This requires the use of a non-cytotoxic ingredient in the simulated fluids. SDS in this respect is less ideal than DPPC and natural surfactants like Curosurf^®^.

### 3.2 Cellular screening

Cytotoxicity screening is the first assessment of drugs for all delivery routes. Generally, this testing uses cell lines relevant for the application and cell number, total protein, total DNA, cellular ATP content, enzyme activity, etc. of exposed cells compared to control cells are common readout parameters. Most often, cellular dehydrogenase activity by metabolization of tetrazolium salts to colored formazan salts is performed (Ekwall et al., 1990; Fröhlich et al., 2009). More specific assays to elucidate the mode of cellular action by assessment of membrane integrity, apoptosis, proliferation, generation of oxidative stress, and organelle function can follow. For cytotoxicity screening of APIs/formulations for pulmonary application various respiratory cells can be used. In general, A549 cells are deemed the most suitable because their CYP enzyme expression pattern is more typical for respiratory cells than that of other cell lines (Castell et al., 2005). A549 cells are derived from an adenocarcinoma of the lung and the phenotype resembles AT2 cells (Shapiro et al., 1978). In case difference between bronchial epithelial cells and alveolar cells is of interest, researchers usually use BEAS-2B bronchial cells. These cells are immortalized bronchial epithelial cells, which have been suggested as representative model for bronchial epithelial cells due the similarity of the expression pattern of metabolizing enzymes (Courcot et al., 2012). More recent data of CYP enzyme expression in BEAS-2B cells, however, confirm the expression pattern only in part (Garcia-Canton et al., 2013). The different cytotoxicity and genotoxicity of multi-walled carbon nanotubes in A549 and BEAS-2B cells may illustrate that bronchial and alveolar epithelial cells react differently (Ursini et al., 2014). The main limitation of BEAS-2B cells is the lack of mucus production. If action of mucus, therefore, is of interest, Calu-3 cells are the most appropriate models because they produce mucus when cultured in the physiologically relevant air-liquid interface culture, where cells are cultured on transwell inserts and supplied with medium only from the basolateral side (Meindl et al., 2015b). Furthermore, Calu-3 cells express metabolizing enzymes similar to BEAS-2B cells and, in addition, are useful indicators for disruption of the cell monolayer because they form a tight epithelial barrier (Foster et al., 2000; Ehrhardt et al., 2008). NuLi-1 cells may be also good models for evaluation of pulmonary effects as they also form tight intercellular junction formation (Molina et al., 2015). However, they capacity for mucus production and expression of CYP enzymes is not clear.

Routine cytotoxicity testing with cells seeded in plastic wells and exposure to different dilutions of the compound is the established procedure for compound screening. It is suggested to include also an assay for membrane integrity as gold standard for cytotoxic action (Niles et al., 2009). In the screening of new formulations physiologically more relevant exposure systems, such as air-liquid interface culture with exposure to the formulation as aerosol or suspended in simulated lung fluid, may improve the predictive value for reaction *in vivo*. In addition to cytotoxicity, evaluation of cellular oxidative stress may be a useful readout parameter as respiratory cells are subjected to higher concentrations of oxygen than other cells in the body. For poorly biodegradable compounds cellular content (accumulation) after repeated dosing of cells might be of interest. Prolonged culture (28 d) of Calu-3 cells in air-liquid interface culture is a way to identify such effects (Fröhlich & Meindl, 2015).

The identification of pro-inflammatory effects triggered either by epithelial cells of the respiratory tract or by AMs is very important. Increased secretion of pro-inflammatory cytokines (for instance IL-6, TNF-α, IL-1β, and IL-8) usually detected by enzyme-linked immunosorbent assay (ELISA), serves as indicator. Additional assays are available to identify adverse effects on Mφ function, for instance on chemotaxis, nitric oxide formation, phagocytosis, and oxidative burst (Prietl et al., 2014). Impairment of phagocytosis is induced when Mφs are exposed to poorly soluble particles, for instance air-borne particulate matter and carbon black particles (Lundborg et al., 2006). This exposure leads to morphological changes of Mφs *in vivo*, usually described as ‘foamy macrophages’, which were then proposed as indicators for adverse effects on Mφs. Similar changes are also observed after exposure to cationic amphiphilic drugs, for instance amiodarone, chloroquine, desipramine, and azithromycin (Shayman & Abe, 2013). According to one theory these drugs form intracellular complexes with phospholipids, which become then resistant to degradation. The cellular changes induce lysosomal fragility and proteolytic enzyme leakage (Forbes et al., 2014). Another theory hypothesizes lysosomal dysfunction as the cause, not the consequence, for the pathological changes (Shayman & Abe, 2013). Formation of the phospholipids may be caused either by inhibition of lipases or by increase of intralysosomal pH. The morphological changes have been termed ‘phospholipidosis’ (PLD) and are characterized by membrane-bound inclusions, primarily lysosomal in origin, with a lamellar structure (‘lamellar bodies’) (Nonoyama & Fukuda, 2008).

In the screening for adverse effects on Mφs mostly murine and rat cells are used. This is due to the fact that immortalized human Mφs are not available and the cells have to be differentiated from monocyte cell lines (THP-1, U937, etc.). Phorbol 12-myristate 13-acetate (PMA) induces the differentiation to Mφs in THP-1 cells, which can further differentiate into M1 and M2 class (Genin et al., 2015). Alternatively, differentiation of monocytes isolated from peripheral blood mononuclear cells (PBMCs) by stimulation with GM-CSF or M-CSF is possible (for instance: (Hassan et al., 1986; Jones et al., 1989; Daigneault et al., 2010)). The differentiation from circulating monocytes with GM-CSF could be an option to study human AMs because monocyte-derived Mφs and AMs isolated from bronchoalveolar fluid of the same individual showed similar rates of phagocytosis while expression of activation surface markers, differed (Forbes et al., 2014). It cannot be excluded that both isolations, AMs from bronchoalveolar lavage and Mφs differentiated from PBMCs, change the original phenotype. It is not possible to decide if data obtained with one of the systems is better than the other because human *in vivo* data for validation are lacking. By comparing Mφ differentiation from PBMCs and from THP-1 cells the authors reported higher cell yield after differentiation from THP-1 cells and higher increase of cell size in Mφs differentiated from PBMCs (Chitra et al., 2014). It is not known to which extent the different sources influence Mφ functions. Cell size, granularity, and surface marker expression are the main parameters for the characterization of the differentiated Mφs. It is assumed that the cell population is not homogenous and contains cells with different extent of differentiation and this heterogeneity may influence the assay results. This heterogeneity can be avoided by the use of murine cell lines because murine Mφs phagocytize particles similarly to human macrophages but behave more homogenous in culture (Gantt et al., 2001).

Murine RAW264.7 cells show morphological changes of PLD upon addition of serum and it is hypothesized that lipoproteins, cytokines and growth factors in the serum trigger these changes. Yao et al. identified micropinocytosis as main mechanism in the formation of lamellar bodies (Yao et al., 2009). In addition to inducing morphological changes, amiodarone impaired phagocytosis of J774A.1 cells, leaving the reaction to endotoxin challenge unchanged (Hoffman et al., 2015). Brasey et al., by contrast, reported only induction of morphological changes in RAW264.7 cells by amiodarone without impairment of phagocytosis (Brasey et al., 2011). It cannot be excluded that cell lines differ in their sensitivity to drug-induced PLD. Phagocytosis has, in any case, been suggested as very sensitive parameter for macrophage impairment (Renwick et al., 2001; Lundborg et al., 2006; Hoffman et al., 2015).

PLD can be detected using lipophilic dyes, such as Oil Red O, Sudan black, Nile red, osmium tetroxide, LipidTox^®^, paraphenylenediamine, etc. that accumulate in lipid-rich organelles (Brown et al., 1992; Hopkins et al., 2010). Vital dyes for lysosome function, namely acridine orange, Lyso-ID^®^, and Lysotracker^®^ are also suitable to identify PLD-inducing drugs like chloroquine (Fröhlich et al., 2012; Meindl et al., 2015a). Furthermore, immunoreactivity against lysosome-associated membrane protein 2 (LAMP2) has been identified as an earlier marker for PLD (Mahavadi et al., 2015).

Various methods are suitable to quantify phagocytosis *in vitro.* Commonly used targets for phagocytosis are fluorescently labeled bacteria (mainly *Escherichia coli*, *Staphylococcus aureus*), IgG-coated and uncoated latex particles, and zymosan (Gu et al., 2014; Kapellos et al., 2016). Quantification is performed by spectrofluorometry, confocal microscopy, flow cytometry, imaging flow cytometry, and automated image analysis. Automated image analysis combines sensitivity with flexibility in magnification, real time kinetics, low cell numbers, and parallel assessment of viability (Kapellos et al., 2016). This techniques, also termed ‘high-content screening’, is the main technology for high-throughput cytotoxicity screening of drug candidates and can also identify changes in cell function or adaptive responses by the evaluation of organelle damage, changes in intracellular signaling, oxidative stress, etc. (Nichols, 2007). One problem in the screening for morphological changes in Mφs is that the clinical relevance of PLD is not entirely clear because >50 drugs, that caused PLD in different tissues, did rarely induce toxicity in patients when taken in prescribed doses (Forbes et al., 2014). This may suggest that pulmonary toxicity only occurs in combination with another stressor. One of the typical inducers of ‘foamy macrophages’ *in vitro*, amiodarone causes pulmonary toxicity in 10–20% of patients (Schwaiblmair et al., 2010). Histologic findings in these patients show morphological changes of PLD in AMs and AT2 cells (Nacca et al., 2012). These findings appear to indicate that screening for effects of inhaled formulations in Mφs has some predictive value for adverse effects in human lungs. Since there is no official FDA policy, drugs that exhibit PLD have been dealt with on a case-by-case basis by industry and FDA.

## 4. Conclusion

Accumulation of APIs at the respiratory barrier, cytotoxicity, and overload of Μφs accompanied by decreased function might be a problem for oral inhalation of drugs. The risk for accumulation is expected to be higher for systemic therapy with higher doses than for the low-dose local medication. In addition to a good physicochemical characterization (mass median diameter, geometric standard deviation, hygroscopicity, zeta potential, etc.) formulations should undergo also *in vitro* biological testing. Given the differences between rodents and human lungs combination of *in vitro* and *in vivo* experiments may improve the value of the preclinical studies. Important *in vitro* screening parameters are particle dissolution, cellular accumulation, cytotoxicity, generation of oxidative stress, and cytokine release in respiratory cells and Mφs, as well as phagocytosis and induction of phospholipidosis in Mφs. Approximate *in vivo* concentrations can be estimated by using the amount of lung lining fluid as distribution volume. APIs and formulations that cause cytotoxicity in the expected dose range should not further be developed. Non-cytotoxic formulations that also do not induce cytokine release and rapidly dissolve in SLF may not need more detailed investigations. For poorly soluble APIs characterization of effects on Mφs may be indicated. These studies should include cellular accumulation, morphological changes, and phagocytosis. In the evaluation of formulations it should be taken into account that not only the API but also excipients can cause the observed adverse effects.
